# Circulating interleukin-18: A specific biomarker for atherosclerosis-prone patients with metabolic syndrome

**DOI:** 10.1186/1743-7075-8-3

**Published:** 2011-01-20

**Authors:** Minako Yamaoka-Tojo, Taiki Tojo, Kazuki Wakaume, Ryo Kameda, Shinji Nemoto, Naonobu Takahira, Takashi Masuda, Tohru Izumi

**Affiliations:** 1 Department of Rehabilitation, Kitasato University School of Allied Health Sciences, 1-15-1 Kitasato, Minami-ku, Sagamihara, 252-0373 Kanagawa, Japan; 2 Kitasato University Graduate School of Medical Sciences, 1-15-1 Kitasato, Minami-ku, Sagamihara, 252-0373 Kanagawa, Japan; 3 Department of Cardioangiology, Kitasato University School of Medicine, 1-15-1 Kitasato, Minami-ku, Sagamihara, 252-0374 Kanagawa, Japan

## Abstract

**Background:**

Metabolic syndrome (MetS) is associated with an increased risk of the development of atherosclerotic cardiovascular disease (CVD). Interleukin-18 (IL-18), which is a pleiotropic proinflammatory cytokine with important regulatory functions in the innate immune response system, plays a crucial role in vascular pathologies. IL-18 is also a predictor of cardiovascular death in patients with CVD and is involved in atherosclerotic plaque destabilization.

**Results:**

In order to determine if circulating levels of IL-18 can serve as a specific biomarker for distinguishing MetS patients from pre-MetS subjects, we studied 78 patients with visceral fat deposition and 14 age-matched control subjects. Increased levels of IL-18 were observed more frequently in patients with MetS than in pre-MetS subjects and were positively associated with waist circumference. Serum levels of IL-18 were significantly reduced by a change in weight caused by lifestyle modifications. There was a significant interaction between waist circumference and serum IL-18 concentration. Weight loss of at least 5% of the body weight caused by lifestyle modification decreased IL-18 circulating levels relative to the reduction in waist circumference and blood pressure, suggesting that this degree of weight loss benefits the cardiovascular system.

**Conclusion:**

IL-18 may be a useful biomarker of the clinical manifestations of MetS and for the management of the risk factors of CVD.

## Background

Obesity and the related metabolic syndrome (MetS) are major public health problems [1] that are associated with an increased risk of the development of atherosclerotic cardiovascular disease (CVD). The mechanism of which may be mediated, at least in part, by increased secretion of proinflammatory cytokines by the adipose tissue [2]. MetS consists of atherogenic dyslipidemia (elevated triglycerides and low high-density lipoproteins [HDLs]), elevated blood pressure and glucose levels, and abdominal obesity with prothrombotic and proinflammatory states [1]. MetS is associated with a 5-fold higher risk of the development of type 2 diabetes and a 2.6- to 3-fold higher risk of the development of CVD [3,4]. The pathophysiology underlying MetS is not well defined, and several investigators have sought to identify a single factor that could explain all of the components of the syndrome. In addition to insulin resistance and/or hyperinsulinemia, investigators have found several biomarkers that are associated with MetS, including leptin [3], catecholamines [5], brain natriuretic peptide (BNP) [6], oxidized low-density lipoprotein (LDL) cholesterol [7], uric acid [8], C-reactive protein (CRP) [3], plasminogen activator inhibitor-1 [3], aldosterone [3], cystatin C [9], and carboxy-terminal prevasopressin (copeptin) [10]. This wide variety of biomarkers highlights the diverse pathophysiological perturbations that occur in MetS [10].

Interleukin-18 (IL-18), which is a pleiotropic proinflammatory cytokine with important regulatory functions in the innate immune response system, plays a crucial role in vascular pathologies. IL-18 is also known as a predictor of cardiovascular death in CVD patients and is involved in atherosclerotic plaque destabilization. A growing body of evidence suggests that IL-18 levels may be closely related to MetS and its consequences [11-13]. Increasing levels of circulating IL-18 have been reported to be closely associated with the components of MetS and to predict type 2 diabetes, cardiovascular events, and mortality [14,15]. IL-18 is secreted constitutively in many different cell types in the adipose tissue, including macrophages, vascular endothelial cells, vascular smooth muscle cells, and adipocytes [16,17]. On the other hand, aerobic exercise has been reported to reduce levels of CRP and IL-18 in subjects with type 2 diabetes [18,19].

We hypothesized that circulating levels of IL-18 may enhance atherosclerosis-prone conditions in patients with MetS. In order to examine this hypothesis, we studied the circulating levels of IL-18 in MetS patients and in subjects with pre-MetS conditions. Furthermore, the circulating levels of IL-18 were examined in MetS patients before and after lifestyle modifications that resulted in weight loss.

## Results and Discussion

### Results

#### Circulating IL-18 levels as a specific biomarker to distinguish MetS patients from subjects with pre-MetS conditions

In order to determine whether the circulating levels of IL-18 could be a specific biomarker to distinguish MetS patients from subjects with pre-MetS conditions, we studied 42 patients with MetS or pre-MetS and 14 control subjects (average body mass index [BMI], 23.3). There were 28 patients diagnosed as having MetS (BMI, 30.9), and the remaining 14 patients were designated as pre-MetS (BMI, 29.6), which was defined as the subjects having only 1 component of the MetS criteria proposed by the Japanese Society of Internal Medicine. The baseline characteristics of the subjects are shown in Tables 1 and 2. Patients with MetS had a higher BMI and waist circumference.

**Table 1:** Characteristics of the study participants

Characteristics	MetS (n = 28)	Pre-MetS (n = 14)	Control (n = 14)
Age (year)	54.8 ± 12.9	58.9 ± 13.0	56.4 ± 10.1
Sex, female (%)	9 (32.1%)	6 (42.9%)	8 (57.1%)
BMI (kg/m^2^)	30.9 ± 7.7*	27.6 ± 3.2	23.3 ± 2.9
Waist circumference (cm)	102.7 ± 13.2*^#^	95.0 ± 7.5*	78.7 ± 7.2

Data are means ± SD. *P < 0.01 for MetS (or pre-MetS) vs. Control. ^#^*P *< 0.01 for MetS vs. pre-MetS. BMI, body mass index; MetS, metabolic syndrome.

**Table 2:** Components of the metabolic syndrome

Components	MetS	Pre-MetS	Control
Plasma glucose (mg/dL)	136 ± 50*	110 ± 12	99 ± 7
Plasma insulin (IU/mL)	8.7 ± 5.6*^#^	6.3 ± 2.7	5.2 ± 1.5
HOMA-IR	2.0 ± 1.1*	1.7 ± 0.7*	1.3 ± 0.4
HDL cholesterol (mg/dL)	48 ± 12*	59 ± 16*	71 ± 16
Triglyceride (mg/dL)	187 ± 25*	160 ± 24*	69 ± 21
Hypertension, n (%)	21 (75.0%)*	5 (35.7%)^#^	3 (21.4%)

Data are means ± SD. *P < 0.01 for MetS (or pre-MetS) vs. Control. ^#^*P *< 0.05 for MetS vs. pre-MetS. HOMA-IR, homeostatis model assessment; HDL, high density lipoprotein; MetS, metabolic syndrome.

As shown in Table 2, fasting plasma glucose levels, plasma insulin levels, a homeostasis model assessment (HOMA-IR), and triglyceride levels increased in both MetS and pre-MetS subjects. Among them, only plasma insulin levels were significantly higher in patients with MetS compared to those who were pre-MetS. These data suggest that more severe hyperinsulinemia may exist in MetS patients compared to subjects with pre-MetS conditions.

**Figure 1:**
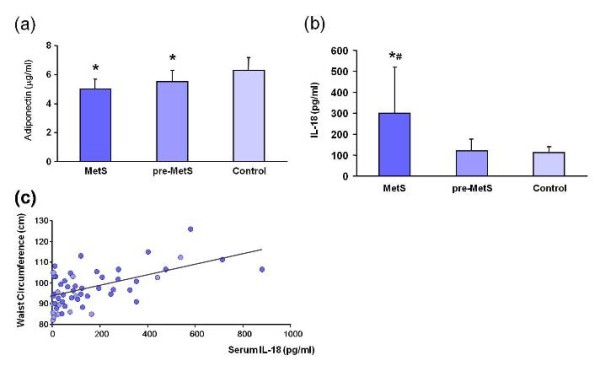
**Circulating Interleukin-18 (IL-18) as a biomarker in the adipocytokine family**. **(a) **Serum levels of adiponectin. *P < 0.05 for MetS (or pre-MetS) vs. Control. **(b) **Circulating levels of IL-18. *P < 0.01 for MetS (or pre-MetS) vs. Control. ^#^*P *< 0.01 for MetS vs. pre-MetS. **(c) **Serum levels of IL-18 and waist circumference. Correlations: *P *< 0.01.

Increased levels of glycated hemoglobin (HbA1c), CRP, and IL-18 were observed in MetS patients (Table 3). Decreased serum adiponectin levels were observed in patients with both MetS and pre-MetS compared with those in control subjects (Figure 1a). There was no difference in adiponectin levels between MetS and pre-MetS patients (Table 3). Increased levels of CRP and IL-18 likely reflect a low-grade systemic inflammation and the development of atherosclerosis.

**Table 3:** Interleukin-18 and related biomarkers

	MetS	Pre-MetS	Control
HbA1c (%)	6.3 ± 1.3*	5.4 ± 0.4	5.0 ± 0.3
CRP (μg/dL)	365 ± 272*	114 ± 98	82 ± 55
adiponectin (μg/mL)	5.0 ± 0.7*	5.5 ± 0.8*	6.3 ± 0.9
IL-18 (pg/mL)	301 ± 220*^#^	121 ± 31	112 ± 29

Data are means ± SD. *P < 0.05 for MetS (or pre-MetS) vs. Control. ^#^*P *< 0.01 for MetS vs. pre-MetS. HbA1c, haemoglobinA1c; CRP, C-reactive protein; IL-18, interleukin-18; MetS, metabolic syndrome.

As shown in Figure 1b, increased levels of IL-18 were observed more frequently in patients with MetS than in those who were pre-MetS (*P *< 0.01), and these levels were positively associated with fasting insulinemia (*P *< 0.05). Interestingly, serum levels of IL-18 were slightly, but significantly, correlated with the waist circumference in patients with MetS and pre-MetS conditions (Figure 1c). These data suggest that IL-18 may reflect visceral fat deposition and insulin resistance. In conclusion, IL-18 may be a useful biomarker of the clinical manifestations of MetS and for the management of the risk factors of CVD.

#### Circulating IL-18 as a useful biomarker for lifestyle modification

We hypothesized that body weight loss from lifestyle modification would improve systemic inflammation in patients with MetS. Serum IL-18 levels were measured in 57 patients with MetS (average BMI, 32.0) before and after they lost at least 5% of their initial weight by lifestyle modification. As shown in Table 4, subjects in the study were typically abdominally obese patients, 74% of the subjects were diagnosed with hypertension, and 61% had diabetes mellitus (DM) and/or impaired fasting glucose (IFG). There was a significant interaction between serum CRP levels and IL-18 levels in patients with MetS (*P *< 0.01).

**Table 4:** Baseline characteristics of the study participants for lifestyle modification

Characteristics	Values	(range)
Age (year)	55.4 ± 13.2	28-76
Sex, female (%)	27 (54.0%)	
Body mass index (kg/m^2^)	31.9 ± 6.2	24-61
Body weight (kg)	84.5 ± 18.6	54.5-138
Waist circumference (cm)	104.5 ± 12.0	85.5-144
Systolic blood pressure (mmHg)	138.7 ± 17.7	
Diastolic blood pressure (mmHg)	78.3 ± 11.8	
Triglyceride (mg/dL)	189.5 ± 121.3	
HDL-cholesterol (mg/dL)	50.1 ± 11.5	
Plasma glucose (mg/dL)	123.4 ± 37.0	
Hypertension (%)	74	
High triglyceride (%)	49	
Low HDL-cholesterol (%)	11	
DM/IFG (%)	61	

Values are means ± SD or presence of comorbidities (%). HDL, high-density lipoprotein; DM, diabetes mellitus; IFG, impaired fasting glucose.

Among all of the subjects undergoing lifestyle modifications, 89% achieved significant reductions in weight, waist circumference, HOMA-IR, and blood pressure after the lifestyle modification was maintained for an average of 22.6 (5.6) weeks. As shown in Table 5, 22-23 weeks of lifestyle modification could significantly reduce obese conditions, including body weight and waist circumference. Systolic blood pressure was also markedly reduced in these subjects (*P *< 0.01).

**Table 5:** Lifestyle modification-induced changes in anthropometric variables

Characteristics	Values	(range)
Δ Body mass index (kg/m^2^)	1.6 ± 1.1^#^	(0.4-5.0)
Δ Body weight (kg)	5.4 ± 4.4^#^	(2.8-33.0)
Δ Waist circumference (cm)	9.6 ± 5.5*	(1.5-24.5)
Δ Systolic blood pressure (mmHg)	15.3 ± 17.5*	
Δ Diastolic blood pressure (mmHg)	12.1 ± 19.1^#^	

Values are means ± SD (range).*P < 0.01 and ^#^*P *< 0.05 for baseline vs. after lifestyle modification in patients with metabolic syndrome.

**Figure 2:**
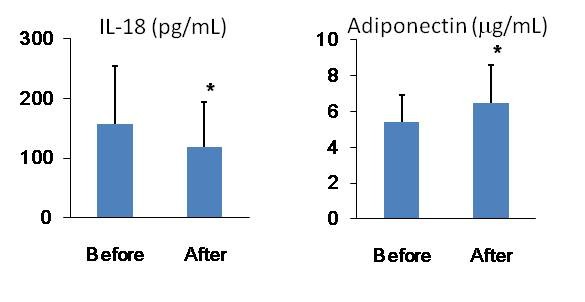
**Circulating levels of interleukin-18 (IL-18) and adiponectin in patients with metabolic syndrome before and after lifestyle modification**. *P < 0.05 for baseline vs. after lifestyle modification in patients with metabolic syndrome.

Serum levels of IL-18 were significantly reduced with weight loss (*P *< 0.01), and the levels significantly correlated with the change in weight (*P *= 0.046). Lifestyle modification-induced weight loss of 5% body weight significantly improved the levels of circulating biomarkers that are related to metabolic and vascular inflammation (Table 6). Most importantly, 5% body weight loss reduced serum levels of IL-18 and synergistically increased serum adiponectin levels in these obese patients (Figure 2).

**Table 6:** Changes in circulating interleukin-18 and related biomarkers by lifestyle modification

	before	after	*P *values
Triglyceride (mg/dL)	190 ± 121	139 ± 67	0.049
HDL-cholesterol (mg/dL)	50 ± 12	56 ± 11	< 0.001
Glucose (mg/dL)	123 ± 37	105 ± 19	0.041
Adiponectin (μg/mL)	5.4 ± 1.5	6.5 ± 2.1	0.034
IL-18 (pg/mL)	158 ± 97	119 ± 75	0.021
CRP (μg/dL)	311 ± 315	204 ± 219	0.012

Data are means ± SD. HDL, high density lipoprotein; IL-18, interleukin-18; CRP, C-reactive protein.

The reduction in IL-18 concentration correlated with increases in adiponectin (*P *= 0.015). A 5% weight loss from lifestyle modification decreased the circulating levels of IL-18 in relation to the reduction in waist circumference, serum CRP levels, and blood pressure, suggesting that this degree of weight loss resulted in cardiovascular benefits.

### Discussion

To the best of our knowledge, this is the first study showing that circulating IL-18 levels can be a useful biomarker for distinguishing patients with MetS from subjects with pre-MetS conditions. Furthermore, a 5% weight loss from lifestyle modification decreased circulating IL-18 levels, a prognostic biomarker for coronary artery disease, in patients with MetS.

#### IL-18 and vascular inflammation

In the last decade, accumulating evidence has suggested the importance of a low but chronic inflammatory state in obesity and in MetS. Recent research has revealed a role of adipose tissue beyond energy storage that involves the harboring of inflammatory cells that are believed to sustain inflammation and impair adipocyte function [20,21]. IL-18 was originally found as an interferon-gamma (IFN-γ)-inducing factor (IGIF) [22] and belongs to the IL-1 family of cytokines [23]. The expression of IL-18 has been reported to be higher in atherosclerotic plaques than in normal control arteries. In addition, IL-18 was found to localize mainly in plaque macrophages and express strongly in unstable plaques [24]. These data suggested that IL-18 plays a major role in atherosclerotic plaque destabilization, which leads to acute ischemic syndromes. In an atherosclerotic animal model, murine IL-18 binding protein (the endogenous inhibitor of IL-18) prevents fatty streak development in the thoracic aorta of apoE knockout mice and slows the progression of advanced atherosclerotic plaques in the aortic sinus [25]. Moreover, IL-18 has been recently shown to contribute to cardiac dysfunction following ischemic reperfusion in vitro [26]. These findings, taken together, identify the inhibition of IL-18 signaling as an important therapeutic target for preventing atherosclerotic plaque development and for inhibiting plaque complications [25].

#### IL-18 and metabolic syndrome

IL-18 concentrations are increased in patients with type 2 diabetes, obesity, and polycystic ovary syndrome [27-29]. In addition, circulating IL-18 has been reported to be closely associated with MetS and its components [30]. Paradoxically, IL-18-deficient mice had markedly increased body weight compared with wild type littermates at 3 months of age, and these mice exhibited obesity, insulin resistance, hyperglycemia, lipid abnormalities, and atherosclerosis. The weight gain was associated with significantly increased body fat; food intake; and glucose, insulin, glucagon, cholesterol, and leptin levels. A histological analysis of various organs of the mutant mice showed only an increased size of the pancreatic islets. Leptin administration or the intracerebral, but not intravenous, administration of recombinant IL-18 reduced food intake. Intraperitoneal administration of recombinant IL-18 restored insulin sensitivity and corrected the hyperglycemia through the activation of signal activation of transcription 3 (STAT3) phosphorylation in IL-18 double-knockout mice. These data suggest that IL-18 has an important role in the homeostasis of energy intake and insulin sensitivity.

In the present study, circulating levels of IL-18 were significantly reduced after lifestyle modification, which resulted in a synergistic reduction in serum CRP levels, plasma glucose levels, and triglyceride levels in patients with MetS. On the other hand, obese patients with type 2 diabetes have been reported to produce significantly less IFN-γ in the peripheral blood mononuclear cells in response to IL-18 stimulation compared to lean controls, which was most likely due to the reduced expression of the IL-18 receptor β chain [31]. This has led to a new concept of IL-18 resistance, which is similar to insulin resistance, in MetS patients. This new concept of IL-18 resistance may shed further light upon the mechanisms involved in the IL-18-related effect on systemic metabolic disorder. At this point, IL-18-mimetic agents or interventions, including lifestyle modifications, may be novel therapeutic strategies for patients with not only pre-MetS conditions but also MetS and atherosclerosis-prone conditions.

IL-18 gene polymorphisms have been shown to be associated with increased levels of circulating IL-18 [32] and one such polymorphism was associated with impaired insulin sensitivity and an increased risk of having MetS [33,34]. These findings suggest that IL-18 may be involved in the pathogenesis of MetS [11]. Therefore, genetic variations of IL-18 influence circulating levels of IL-18 and the clinical outcome in patients with coronary artery disease.

#### IL-18 as a predictive biomarker for cardiovascular events

In previous studies in patients with documented coronary artery disease, serum IL-18 levels were elevated in patients with acute coronary syndrome [35] and were a strong independent predictor of cardiovascular death [14,36]. In a previous cohort study, circulating IL-18 was the only independent predictor of cardiovascular mortality in a subgroup with MetS [37]. Moreover, circulating IL-18 levels were a strong and independent predictor of cardiovascular events in elderly men with MetS [38]. Increased levels of IL-18 were associated with the presence of subclinical atherosclerosis, which was evaluated by an examination of the intima media thickness of the carotid artery [39], and by measurement of arterial stiffness, which was determined on the basis of brachial pulse wave propagation [40], after adjustment for traditional risk factors. However, others have reported that circulating IL-18 levels were associated with carotid media thickness in univariate analyses, but not after adjustment for traditional risk factors [13,41] or in multiple analyses [42].

#### IL-18 as a therapeutic biomarker to enhance coronary risk factor management in MetS patients

A growing body of evidence supports an important role of IL-18 in the pathogenesis of MetS and atherosclerosis. In this study, a 5% weight loss from lifestyle modification decreased circulating levels of IL-18 and CRP. According to the results of previous clinical studies, weight loss mediated by calorie-restricted diet interventions [43], Mediterranean-like diets, and omega-3 fatty acid supplementation [44], or combined interventions with diet and exercise [45,46], were reported to decrease IL-18 levels [40]. Aerobic exercise has been reported to reduce circulating levels of IL-18 in patients with type 2 diabetes [18,19] and IL-18 expression in adipose tissue in obese subjects [47].

### Study limitations

A limitation of the current study is that the study cohort was small. Since the circulating levels of IL-18 correlated well with the waist circumference in patients with MetS and subjects with pre-MetS conditions, it is reasonable to speculate that there may have also been a difference detected between the MetS and pre-MetS groups, if the group sizes were bigger. However, the group sizes for the examination of the effects of lifestyle modification were adequate to reveal significant differences in the changes in the circulating levels of IL-18 and other inflammatory biomarkers.

## Conclusions

In summary, higher circulating levels of IL-18 were associated with increased MetS scores and systemic inflammation, which was independent of the presence of diabetes or dyslipidemia. Circulating IL-18 may be a novel biomarker for high-risk patients with MetS, and further studies are warranted in order to assess its utility as a predictor of the presence of MetS and atherogenic conditions. These findings suggest that IL-18 dysfunction or resistance is a novel pathophysiological mechanism underlying insulin resistance and MetS. Moreover, a 5% weight loss from lifestyle modification decreased the circulating levels of IL-18 relative to the reduction in waist circumference, vascular inflammation, and blood pressure, suggesting that this degree of circulating IL-18-guided weight management may be effective for cardiovascular benefits and the prevention of cardiovascular events.

## Materials and methods

### Subjects

The study included 78 Japanese outpatients with abdominal obesity (waist circumference, >85 cm for men and >90 cm for women) who consulted for medical care in the Metabolic Syndrome Clinic, Department of Cardioangiology, Kitasato University Hospital. All subjects provided informed consent before participating in this study, and anonymity was maintained by tracing the patients through their clinical history number.

The project was approved by the Scientific and Ethical Committee of the Kitasato University School of Medicine, Japan. BMI was calculated as weight divided by height squared. Systolic and diastolic blood pressures were measured after a rest of at least 15 min with a sphygmomanometer while subjects were in a sitting position. HOMA-IR, which was used as a measure of insulin resistance, was calculated as fasting plasma insulin (μU/mL) × glucose (mg/dL)/405 [48]. All subjects were free from chronic inflammation, immune disease, acute coronary syndrome, renal and/or liver dysfunction, malignancy, or immune diseases.

### Definitions of MetS and pre-MetS

Metabolic scores were calculated using MetS components according to the MetS criteria proposed by the Japanese Society of Internal Medicine [49]. The score consisted of 4 independent components, including abdominal obesity, which was defined as a waist circumference of ≥85 cm in men or ≥90 cm in women; high triglyceride and/or low HDL-cholesterol levels; hypertension; and elevated fasting glucose levels. The diagnosis of hypertension was made on the basis of blood pressure levels measured at the study visit (≥130/85 mmHg) or a prior diagnosis of hypertension and current treatment with antihypertensive medications. DM and/or IFG was considered present if the subject had a history of diabetes or had a fasting glucose level of 110 mg/dL or greater. Participants who had low metabolic scores (1 or 2) were designated as the pre-MetS subjects, whereas the patients who had high metabolic scores (3 or 4) were defined as MetS subjects. There were 64 patients diagnosed as having MetS and the remaining 14 patients were designated as pre-MetS, which was defined as having only 1 component of the MetS criteria.

### Serum Sample Collection

After an overnight fast, blood serum samples were collected by venipuncture from 78 patients with abdominal obesity and from 14 healthy donors. The age range of the healthy donors matched that of the patients. For the IL-18 biomarker study examining the differences in IL-18 levels between MetS patients and subjects with pre-MetS conditions, 36 patients out of the 64 patients with MetS were excluded because they were taking metabolic-mimetic agents (statins, anti-diabetic drugs, and/or colestimide).

### Measurement of Clinical Biomarkers

Biochemical markers such as triglycerides, LDL cholesterol, HDL cholesterol, insulin, plasma glucose, HbA1c, uric acid, gamma-glutamyl transpeptidase (γ-GTP), CRP, and BNP were measured.

### Lifestyle Modification

In order to examine the effects of lifestyle modification in the IL-18 biomarker study, 64 patients with MetS were recruited to lose weight in order to improve their cardiovascular risk factors. We studied 57 patients with MetS (average BMI, 31.9) who successfully lost at least 5% of their initial weight through lifestyle modification. All subjects were instructed to maintain a standard mild energy-restricted diet and to engage in walking for at least 30 min a day, 5 days a week. Serum IL-18 and adiponectin levels were measured in these subjects before and after lifestyle modification, which was maintained for 8-34 weeks.

### Measurement of Adipocytokines

Circulating levels of human IL-18 and adiponectin were determined by an enzyme-linked immunosorbent assay (ELISA) using the human IL-18 ELISA Kit (MBL, Co., Ltd., Nagoya, Japan) and the CircuLex™ human adiponectin ELISA Kit (CycLex Co., Ltd., Nagano, Japan), respectively. Samples were processed according to the manufacturer's instructions [35,50].

### Statistical Analysis

Continuous data are summarized as either mean (SD) or median and quartiles, and categorical data are expressed as percentages. The data were compared by an unpaired *t*-test or Mann-Whitney *U*-tests, where appropriate. Differences in the proportions of variables were determined by a chi-squared analysis. In order to evaluate the relationship between IL-18 and selected variables, we calculated Spearman correlation coefficients between the circulating levels of fasting IL-18 and the following variables: (1) conventional risk factors for CVD (i.e., LDL cholesterol, HDL cholesterol, triglycerides, HbA1c, the presence of hypertension, and current smoking status), and history of coronary artery disease; (2) measures of adiposity and insulin resistance (i.e., BMI, waist circumference, fasting blood glucose levels, insulin levels, and HOMA-IR); and (3) metabolic risk scores (i.e., abdominal obesity, hypertension, high triglycerides and/or low HDL cholesterol, and glucose intolerance or diabetes) and MetS-related co-morbid conditions (i.e., hyper-uric acidemia, fatty liver disease, chronic kidney disease, and sleep apnea syndrome).

In order to evaluate the association of IL-18 with MetS, we constructed multivariable logistic regression models in order to assess whether the circulating IL-18 levels were independently associated with MetS. We calculated the odds ratio for the presence of MetS in each IL-18 level quartile, and the participants with IL-18 levels in the lowest IL-18 quartile were considered as the reference group. Adjustments were performed for age and sex and for age, sex, and β-blocker use. Two-sided *P*-values of < 0.05 were considered significant.

## Competing interests

The authors declare that they have no competing interests.

## Authors' contributions

MY-T participated in the design of the study and performed the statistical analysis. TT conceived of the study, and participated in its design and coordination and helped to draft the manuscript. Other authors participated in enrolling patients in the study and discussion. All authors read and approved the final manuscript.
